# Morphological, functional and biochemical differences in cardiac adaptation to endurance exercise among male and female amateur marathon runners

**DOI:** 10.3389/fphys.2025.1547894

**Published:** 2025-03-04

**Authors:** Zofia Lasocka-Koriat, Zuzanna Lewicka-Potocka, Anna Kaleta-Duss, Nikola Bulman, Ewelina Marciniak, Leszek Kalinowski, Ewa Lewicka, Alicja Dąbrowska-Kugacka

**Affiliations:** ^1^ Department of Cardiology and Electrotherapy, Medical University of Gdańsk, Gdańsk, Poland; ^2^ First Department of Cardiology, Medical University of Gdańsk, Gdańsk, Poland; ^3^ Institute for Radiology, Cantonal Hospital Aarau, Aarau, Switzerland; ^4^ Department of Medical Laboratory Diagnostics—Fahrenheit Biobank BBMRI.pl, Medical University of Gdańsk, Gdańsk, Poland; ^5^ Department of Mechanics of Materials and Structures, BioTechMed Centre, Gdańsk University of Technology, Gdańsk, Poland

**Keywords:** sports cardiology, amateur athletes, echocardiography, cardiac biomarkers, sex-related remodelling

## Abstract

**Introduction:**

Sport is known to have beneficial influence on cardiovascular system. However, activities of high intensity such as marathon running may adversely affect cardiac morphology and function, especially in the heterogenous group of amateur athletes. As males and females exhibit discrepancies in cardiac response to training, we aimed to compare exercise-induced myocardial alterations between sexes among 61 amateur marathon runners, with the use of evolving echocardiographic techniques and cardiac biomarkers.

**Methods:**

The study followed three stages: 2–3 weeks prior the marathon (Stage 1), at the finish line (Stage 2) and 2 weeks after the run (Stage 3). Echocardiographic examination along with blood analyses for biomarkers of cardiac injury and overload [creatine kinase, high sensitivity cardiac troponin I, heart-type fatty acid binding protein, B-type natriuretic peptide, galectin-3 (Gal-3), endothelin-1 (ET-1), interleukin-6 and neopterin] were performed at each stage.

**Results:**

After the marathon there was a transient increase in right ventricular (RV) size and concomitant decrease in left ventricular (LV) volumes, leading to a significant increase of RV end-diastolic volume (RVEDV)/LVEDV ratio (0.91 ± 0.21 vs. 1.10 ± 0.22, p < 0.001 in males; 0.73 ± 0.17 vs. 1.02 ± 0.22, p < 0.001 in females). Although at Stage 2 RV contractility decreased, while LV ejection fraction (LVEF) remained at the same level in both sexes, men had greater tendency for LVEF reduction (p < 0.05 for the interaction sex and stage). The concentrations of biomarkers were higher after the run in both study groups, except for ET-1 and neopterin, which increased post-race only in males. The larger training-related rise in Gal-3 level correlated with the greater drop in LVEF at Stage 2 (r = −0.42; p < 0.05). Less-trained marathoners with lower VO_2_max values after the race showed higher levels of Gal-3 post-run (r = −0.29; p < 0.05).

**Conclusion:**

Marathon running induces transient cardiac remodelling, more pronounced in male than female athletes. Structural and functional changes assessed by echocardiography correspond with biochemical alterations. Galectin-3 was the best biomarker to reflect overload changes. Cardiovascular screening in amateur runners should be implemented to identify subjects requiring further evaluation.

## 1 Introduction

Cardiovascular adaptations to exercise include structural, functional and biochemical changes that differ with respect to the type of training, ethnicity, age and sex (Maron and Pelliccia, 2006). Endurance sports, such as marathon running, have involved increasing number of participants yearly, mainly due to the higher rate of amateur athletes that engage in sport for pleasure but present with a broad spectrum of lifestyle risk factors (Vitti et al., 2020). In addition, the growing popularity of long-distance running is considered more pronounced in women than men. Indeed, the number of female athletes participating in the “Berlin-Marathon” has increased from only 8 in 1974 to above 12 thousand in 2019 (Reusser et al., 2021). As sports science research has been dominated by investigations on males, with only 4% of studies conducted exclusively on female counterparts (Science News, 2016), it is important to enrich the existing pool of knowledge with more data on female athletes, especially amateur marathon runners.

Regular physical training has been shown to reduce the risk of cardiovascular diseases and, consequently, all-cause mortality, increasing the average life expectancy (Paffenbarger et al., 1986). However, repeated bouts of endurance exercise associated with transient volume and pressure overload are known to cause adaptative changes in cardiac morphology and function, which may eventually mimic certain pathological conditions. Men and women exhibit different cardiac response to training. Female athletes adapt primarily by increasing chambers’ dimensions, while in males concentric cardiac remodelling predominates (Finocchiaro et al., 2017). The structural alterations usually correspond with slight or moderate impairment in function, to a greater extent in men than in women (D'Ascenzi et al., 2020). There are several factors that determine sex-specific cardiac response to exercise, including hormonal profile, molecular and genetic mechanisms (Colombo and Finocchiaro, 2018).

Estrogen in females has a protective effect on cardiomyocytes, attenuating adverse cardiac remodelling, while testosterone in males induce hypertrophy by increasing cardiac protein synthesis (Patrizio and Marano, 2016). Moreover, the higher levels of circulating estrogen in females contribute to discrepancies in substrate oxidation during exercise. Women are thought to have enhanced adaptive lipolytic activity and, therefore, derive more energy from fatty acids, whereas men base upon the carbohydrate oxidation (Horton et al., 1985). As most of available research focuses on premenopausal females, it is noteworthy that menopause causes a decline in plasma estrogen concentrations, influencing cardiac adaptation to training in female athletes.

Sex differences exist also in the neuroendocrine response during exercise (Scott et al., 1985). Endurance training is associated with an increased sympathetic activation in men with higher systolic and mean arterial pressures. On the contrary, women have greater parasympathetic drive and lower sympathetic control of heart rate (Davis et al., 2000). Taking genetic factors into consideration, further sex-specific discrepancies regard gene polymorphisms involved in the renin-angiotensin-aldosterone system, responsible for the development of left ventricular (LV) hypertrophy (Min et al., 2009). However, additional investigations of the mechanisms through which gender regulates cardiac response to exercise should be performed in order to increase the applicability of these data in the clinical settings.

Along with cardiac volumetric and functional adaptations detected by visual imaging techniques, including modern speckle tracking echocardiography (STE) or real-time, three-dimensional (3D) echocardiography, biochemical blood analysis should not be underestimated. Numerous studies revealed that endurance training results in transient elevation in concentrations of cardiac biomarkers both in male and female athletes (Scharhag et al., 2008; Frassl et al., 2008; Kaleta-Duss et al., 2020). However, the question arises whether the increase in any given parameter represents clinically significant cardiac injury, or is just part of physiological response to exercise. What is more, apart from biomarkers routinely used in daily practice, there are several novel parameters, such as heart-type fatty acid binding protein (H-FABP), galectin-3 (Gal-3), endothelin-1 (ET-1) and proinflammatory cytokines, of growing importance in cardiovascular screening. Although these biomarkers are not pathognomonic for cardiomyocyte damage, fluctuations in their concentrations depending on the training load may predict outcomes in athletes.

Therefore, the purpose of our study was to compare the influence of marathon run on cardiac remodelling between male and female amateur athletes, with the use of evolving echocardiographic techniques and cardiac biomarkers. In addition, we aimed to investigate the potential correlations between structural, functional and biochemical changes that determine sex-specific cardiac response to endurance exercise. Finally, it might be considered that acute bout of training, such as marathon run, results in corresponding adaptative alterations in elite and amateur athletes.

## 2 Materials and methods

### 2.1 Study design and participants

We recruited 67 amateur marathon runners of both genders, aged between 24 and 57 years. A total of 40 male participants were enrolled from the 2nd PZU Gdansk Marathon in 2016, while 27 females from the XXIV Orlen Solidarity in 2018 and 5th Gdansk Marathon in 2019. All athletes were healthy, in sinus rhythm, without cardiovascular comorbidities or other chronic diseases. In the pre-participation screening, training routine and family history of cardiac disease or sudden cardiac death (SCD) were collected from every volunteer.

The study was divided into three stages: 2–3 weeks before the marathon start (Stage 1), immediately after the race, on the finish line (Stage 2), and 2 weeks after the competition (Stage 3). Each stage included physical examination with anthropometric measurements, electrocardiographic (ECG) and echocardiographic assessment, along with blood samples’ collection for further biochemical analysis. Additionally, at baseline all amateur marathon runners underwent cardiopulmonary exercise test (CPET) on a treadmill in order to assert their exercise capacity, and 24-h Holter ECG monitoring to detect potential arrhythmias. During the competition, participants were allowed to rehydrate on a whim, and no food intake restrictions were advised.

The study protocol was approved by the Independent Bioethics Commission for Research of the Medical University of Gdansk (NKBBN/104/2016), and all participants gave written informed consent.

### 2.2 Cardiopulmonary exercise test

Ergospirometry was performed on the treadmill (H/P/Cosmos Saturn treadmill) using the Bruce protocol. First stage started at 2.7 km/h and at 10% gradient, then the speed and incline were increased in 3 min intervals. Jaeger OxyconPro equipment with Jlab Manager V5.32.0 software was used to measure the oxygen uptake (VO_2_), carbon dioxide output (VCO_2_), minute ventilation (VE), expiratory gas concentrations throughout the respiratory cycle on a breath-by- breath basis. The maximum oxygen uptake (VO_2_max) was calculated as the highest oxygen volume averaged over 10 s at peak work load.

### 2.3 Echocardiographic assessment

Standard and 3D transthoracic echocardiography was performed using a commercially available system (Vivid E9 and E95, GE Healthcare, Horten, Norway), equipped with a 4VD transducer. All participants were positioned in the left-lateral decubitus position and examined by experienced sonographers, in accordance with the current guidelines of the American Society of Echocardiography (ASE) and European Association of Cardiovascular Imaging (EACVI) (Lang et al., 2015). The obtained views were analysed off-line using dedicated quantification software (EchoPac 201, GE Healthcare, Norway).

For the real-time 3D echocardiography, an ECG-triggered multiple-beat full-volume data set of both ventricles was acquired from the apical view from six cardiac cycles during an end-expiratory breath-hold. The blood–tissue interface was automatically initialized by the software and afterwards manually corrected frame-by-frame by tracing the endocardium from the 2-, 3-, and 4-chamber and short-axis views. To assess 3D morphology of LV and right ventricle (RV), we determined ventricular end-diastolic volume (EDV) and ventricular end-systolic volume (ESV), indexed to body surface area (BSA). Although it would have also been possible to scale by the method based on the similarity of the exponents, in this study the most frequent technique was applied. The obtained values were used to calculate RVEDV/LVEDV ratio, so as to evaluate interventricular interactions. Analysis of LV systolic function included indexed LV stroke volume (LVSV) and LV ejection fraction (LVEF). While in the case of RV, RV ejection fraction (RVEF) was assessed.

The diastolic function of LV and RV was assessed with pulsed wave Doppler (PWD) and spectral Doppler tissue imaging (DTI) from the apical 4-chamber view. Transmitral and transtricuspid PWD inflows at the tips of valve leaflets were measured to obtain peak early (E) and peak atrial (A) flow velocities, E/A ratio and the deceleration time (DT) of the E-wave velocity. DTI of septal and mitral lateral annulus was obtained, and an average peak early diastolic (e’) value was used. Subsequently, E/e’ ratio was calculated. The position of the sample volume for velocity and strain measurements was manually set in the myocardium throughout the cardiac cycle.

### 2.4 Biochemical assessment

The study protocol included three blood samples obtained from the cubital veins of amateur runners. The blood was collected 2 weeks before the marathon, just after finishing the run, and 2 weeks after the race. At Stage 1 and Stage 3, fasting blood samples were gathered. None of the samples showed any signs of hemolysis. Serum was obtained by centrifugation at 2000 rpm at room temperature for 12 min. Aliquots were stored at −80°C for later analyses. Total creatine kinase (CK) was measured by optimized spectrophotometric method with the Cobas 6000 (Roche Diagnostics) analyzer. Serum high sensitivity cardiac troponin I (hs-TnI) levels were calculated using Architect I2000 (Abbott) analyzer. H-FABP, B-type natriuretic peptide (BNP), Gal-3, ET-1, interleukin-6 (IL-6) and neopterin concentrations were determined by the enzyme immunoassay method using commercially available kits (H-FABP, Gal-3, ET-1, IL-6 – R&D Systems, Minneapolis, USA; BNP–Wuhan EIAab Science Co.,Ltd., China; Neopterin–Demeditec Diagnostics GmbH, Kiel, Germany).

### 2.5 Statistical analysis

All statistical analyses were conducted using the licensed Statistica 13.3 software (Statsoft Inc., Tulsa, Oklahoma, United States). Shapiro–Wilk test was applied to differentiate between normally and non-normally distributed variables. The continuous data were presented as mean ± standard deviation (SD). For each parameter 95% confidence interval (CI) of the mean was calculated. To evaluate changes over three stages of the study along with sex*stage interaction we performed ANOVA analysis and the *post hoc* Tukey test for normally distributed data, or a Friedman test and *post hoc* test for non-normally distributed variables. Spearman and Pearson’s correlations were calculated to determine the relationship between echocardiographic findings and concentration of indicated cardiac biomarkers. The p-value of < 0.05 was considered statistically significant.

## 3 Results

### 3.1 Study group

The study included 67 Caucasian amateur marathon runners. Among them, 61 sportsmen successfully completed the race, and underwent both echocardiographic and biochemical examination – 34 male (mean age 39 ± 9 years) and 27 female (mean age 40 ± 7 years) amateur athletes. The baseline characteristics of the study population with morphometric data are presented in Table 1. The weight, height, body mass index (BMI), BSA and VO_2_max were significantly higher in males than females. Comparing the intensity of training of the study participants, female athletes had larger mean training time per week, while no significant differences were detected in the training distance per week between sexes. The mean marathon finishing time was shorter in males.

**TABLE 1:** Physical characteristics and intensity of training of the studied group.

Variable	Male (N = 34)	Female (N = 27)	p-value
	Mean ± SD		
Age, years	39 ± 9	40 ± 7	0.623
Weight, kg	80 ± 7	59 ± 8	<0.001
Height, cm	179 ± 6	166 ± 5	<0.001
BMI, kg/m^2^	25 ± 2	22 ± 3	<0.001
BSA, m^2^	2.0 ± 0.1	1.7 ± 0.1	<0.001
VO_2_max, mL/kg/min	53.6 ± 6.6	42.8 ± 5.1	<0.001
Training intensity			
hours of running/week	6.5 ± 2.2	8.1 ± 3.5	0.039
distance running/week, km	56.3 ± 19.6	60.7 ± 27.8	0.478
Marathon performance time, min	234 ± 23	253 ± 33	0.009

SD, standard deviation; BMI, body mass index; BSA, body surface area; VO_2_max, maximal oxygen uptake.

Holter monitoring, performed at baseline, revealed the underlying sinus rhythm with an average heart rate (HR) of 67.4 ± 9.0 bpm in male and 64.0 ± 6.3 bpm in female athletes. There were no remarkable arrhythmias in any of the study groups. Only the number of premature ventricular contractions (PVCs) per day was slightly higher in females compared to males (2.8 ± 6.4 vs. 0.1 ± 0.3; p = 0.014).

### 3.2 Echocardiographic measurements

**FIGURE 1:**
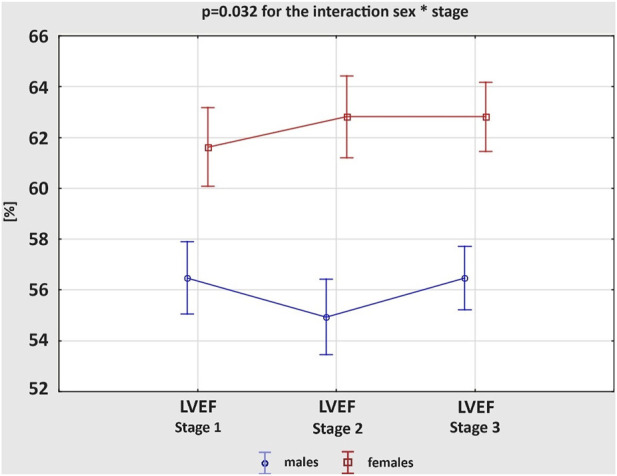
Changes in the left ventricular ejection fraction (LVEF) between three study stages in male and female amateur marathon runners.

Table 2 presents exercise-induced 3D echocardiographic parameters of LV and RV in male and female amateur marathon runners. The comparison of results from Stages 1 and 3 revealed no differences in any of the studied groups. Analysing morphological changes of ventricles, female athletes had significantly larger LVEDV index than male counterparts both at rest and after the run, while RV volumes did not differ remarkably between sexes at any stage of the study. Consequently, the calculated RVEDV/LVEDV ratio was proved greater in the male group, reaching statistical significance at baseline before the competition start. After the marathon, we noticed a reduction in LV volumetric parameters, such as LVEDV, LVESV and LVSV, and concomitant increase in RV volumes, leading to a significant growth of RVEDV/LVEDV ratio. This observation was compatible in male and female amateur athletes. The systolic function of LV and RV achieved larger values among female runners at Stage 1 and 2. The marathon run resulted in significant deterioration of RV contractility, with negligible influence on LVEF in both sexes. However, the direction of post-race changes in LVEF differed between genders; p = 0.032 for the interaction sex * stage (Figure 1) – it slightly increased in females and decreased in males, resulting in a significant discrepancy.

**TABLE 2:** Three-dimensional echocardiographic measures of left and right ventricle in male and female amateur athletes.

Parameter	Male (N = 34)					Female (N = 27)					Males vs. Females	
	Stage 1	Stage 2	Stage 3	S1 vs. S2 p-value	S1 vs. S3 p-value	Stage 1	Stage 2	Stage 3	S1 vs. S2 p-value	S1 vs. S3 p-value	Stage 1	Stage 2
	Mean ± SD (95% CI)					Mean ± SD (95% CI)					p-value	
LVEDV index, mL/m^2^	57.2 ± 10.4 (53.5–60.9)	52.0 ± 8.7 (48.9–55.1)	57.2 ± 11.0 (53.3–61.1)	**<0.001**	>0.05	66.2 ± 7.9 (63.1–69.4)	60.1 ± 9.5 (56.4–63.9)	68.1 ± 8.5 (64.8–71.5)	**<0.001**	>0.05	**<0.001**	**0.001**
LVESV index, mL/m^2^	25.0 ± 5.3 (23.1–26.9)	23.6 ± 4.2 (22.0–25.1)	25.0 ± 5.2 (23.1–26.8)	**0.036**	>0.05	25.4 ± 4.3 (23.8–27.1)	22.4 ± 4.2 (20.7–24.0)	25.5 ± 4.4 (23.8–27.2)	**<0.001**	>0.05	—	—
LVSV index, mL/m^2^	32.2 ± 5.8 (30.2–34.3)	27.8 ± 4.4 (26.2–29.4)	32.3 ± 6.5 (30.0–34.6)	**<0.001**	>0.05	40.8 ± 5.8 (38.5–43.1)	36.9 ± 7.5 (33.9–39.8)	42.6 ± 6.3 (40.1–45.1)	**<0.001**	>0.05	**<0.001**	**<0.001**
LVEF, %	56.4 ± 3.6 (55.1–57.7)	54.9 ± 4.6 (53.3–56.6)	56.4 ± 3.6 (55.1–57.6)	—	—	61.6 ± 4.5 (59.9–63.4)	62.8 ± 3.7 (61.4–64.3)	62.8 ± 3.6 (61.4–64.2)	—	—	**<0.001**	**<0.001**
RVEDV index, mL/m^2^	50.7 ± 11.3 (46.6–54.7)	56.2 ± 9.9 (52.5–59.8)	52.0 ± 9.0 (48.7–55.3)	**0.004**	>0.05	48.8 ± 11.0 (43.8–52.4)	60.0 ± 11.1 (55.7–64.4)	48.3 ± 10.9 (43.9–52.7)	**0.001**	>0.05	—	—
RVESV index, mL/m^2^	24.8 ± 6.3 (22.6–27.2)	30.7 ± 6.5 (28.3–33.0)	25.6 ± 5.0 (23.7–27.5)	**<0.001**	>0.05	22.2 ± 6.5 (19.4–24.4)	30.7 ± 7.0 (28.0–33.5)	21.7 ± 5.9 (19.3–24.0)	**<0.001**	>0.05	—	—
RVSV index, mL/m^2^	26.6 ± 6.0 (24.4–28.7)	26.2 ± 4.1 (24.7–27.7)	27.5 ± 6.2 (25.3–29.7)	—	—	26.6 ± 6.4 (23.7–28.8)	29.3 ± 6.1 (27.2–32.0)	26.6 ± 8.1 (23.4–29.8)	—	—	—	**0.014**
RVEF, %	51.1 ± 3.4 (49.9–52.3)	45.7 ± 4.1 (44.2–47.2)	50.5 ± 4.4 (49.0–52.1)	**<0.001**	>0.05	54.9 ± 6.3 (52.4–57.4)	49.1 ± 6.3 (46.6–51.6)	53.9 ± 5.1 (51.9–55.9)	**0.006**	>0.05	**0.004**	**0.015**
RVEDV/LVEDV ratio	0.91 ± 0.21 (0.83–0.98)	1.10 ± 0.22 (1.02–1.18)	0.92 ± 0.27 (0.83–1.02)	**<0.001**	>0.05	0.73 ± 0.17 (0.66–0.80)	1.02 ± 0.22 (0.93–1.10)	0.72 ± 0.19 (0.64–0.80)	**<0.001**	>0.05	**0.001**	—

SD, standard deviation; CI, confidence interval; LVEDV, left ventricular end-diastolic volume; LVEF, left ventricular ejection fraction; LVESV, left ventricular end-systolic volume; LVSV, left ventricular stroke volume; RVEDV, right ventricular end-diastolic volume; RVEF, right ventricular ejection fraction; RVESV, right ventricular end-systolic volume; RVSV, right ventricular stroke volume. Statistically significant values are marked with bold.

**FIGURE 2:**
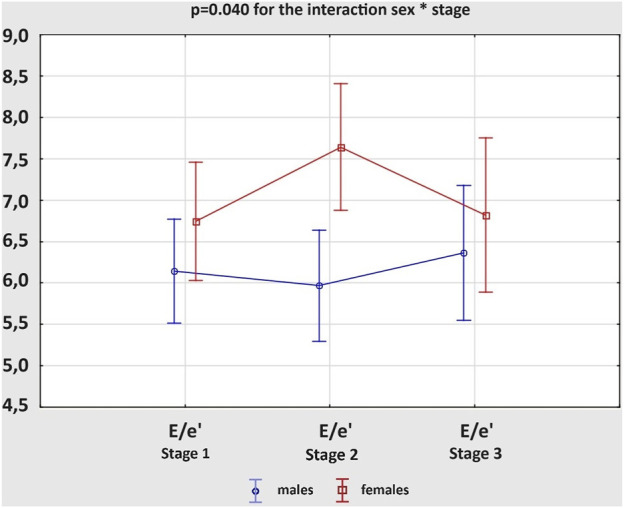
Changes in the *ratio* of peak early mitral inflow velocity to average peak early diastolic mitral tissue velocity (E/e’) between three study stages in male and female amateur marathon runners.

The results on the diastolic function of LV and RV in males and females are shown in Table 3. The average values of the calculated variables were within the normal ranges, regardless of the study group. However, both E− and A-waves of mitral valve (MV) had higher velocities in female athletes than male participants at Stage 1 and 2, resulting in similar E/A ratios. The e’ values were not influenced by sex, while E/e’ ratios were increased in women, significantly only after the marathon. There were no remarkable differences in DT of MV between the groups at any stage of the study. Analysing the acute changes, we observed a significant reduction in the E-wave velocities, along with an increase in the A-wave velocities after the run in both male and female amateur athletes, which resulted in significantly decreased E/A ratios. The e’ values decreased post-race in male and female groups. Although there were no remarkable differences in E/e’ ratio between the stages of the study, female athletes experienced more pronounced growth of the parameter; p = 0.040 for the interaction sex * stage (Figure 2). The exercise-induced reduction in DT of MV reached statistical significance only among women. Comparing diastolic function of RV, males and females presented similar outcomes, regardless of the study stage. However, competing the race caused a significant increase in the A-wave velocities of tricuspid valve (TV), with concomitant decrease in E/A ratios, and reduction in DT of TV. These changes were present among men and women. All of the observed alterations in diastolic parameters of LV and RV were transient and normalized within 2 weeks of detraining, at Stage 3.

**TABLE 3:** Two-dimensional echocardiographic measures of biventricular diastolic function in male and female amateur athletes.

Parameter	Male (N = 34)					Female (N = 27)					Males vs. Females	
	Stage 1	Stage 2	Stage 3	S1 vs. S2 p-value	S1 vs. S3 p-value	Stage 1	Stage 2	Stage 3	S1 vs. S2 p-value	S1 vs. S3 p-value	Stage 1	Stage 2
	Mean ± SD (95% CI)					Mean ± SD (95% CI)					p-value	
MV E, m/s	0.75 ± 0.14 (0.70–0.80)	0.66 ± 0.14 (0.61–0.72)	0.80 ± 0.17 (0.74–0.86)	**0.014**	>0.05	0.85 ± 0.20 (0.77–0.93)	0.76 ± 0.16 (0.68–0.82)	0.85 ± 0.21 (0.76–0.93)	**0.015**	>0.05	**0.028**	**0.030**
MV A, m/s	0.51 ± 0.10 (0.48–0.55)	0.65 ± 0.14 (0.59–0.70)	0.54 ± 0.10 (0.51–0.58)	**<0.001**	>0.05	0.57 ± 0.17 (0.50–0.64)	0.79 ± 0.17 (0.72–0.87)	0.58 ± 0.14 (0.52–0.65)	**<0.001**	>0.05	—	**0.002**
MV E/A	1.51 ± 0.38 (1.37–1.65)	1.07 ± 0.29 (0.96–1.18)	1.52 ± 0.37 (1.39–1.65)	**<0.001**	>0.05	1.63 ± 0.71 (1.33–1.92)	0.97 ± 0.20 (0.88–1.07)	1.49 ± 0.53 (1.25–1.73)	**<0.001**	>0.05	—	—
MV e’, m/s	0.13 ± 0.02 (0.12–0.13)	0.11 ± 0.02 (0.10–0.12)	0.13 ± 0.03 (0.12–0.14)	**0.004**	>0.05	0.14 ± 0.02 (0.13–0.14)	0.10 ± 0.02 (0.09–0.11)	0.14 ± 0.03 (0.13–0.15)	**<0.001**	>0.05	—	—
MV E/e’	6.19 ± 1.38 (5.68–6.70)	5.95 ± 1.31 (5.44–6.46)	6.45 ± 1.85 (5.79–7.11)	—	—	6.51 ± 1.70 (5.84–7.19)	7.54 ± 1.96 (6.63–8.46)	6.52 ± 2.03 (5.68–7.36)	—	—	—	**<0.001**
MV DT, ms	194 ± 40 (179–209)	182 ± 50 (163–201)	186 ± 49 (168–203)	—	—	197 ± 42 (179–214)	163 ± 40 (143–184)	191 ± 36 (175–207)	**0.029**	>0.05	—	—
TV E, m/s	0.52 ± 0.08 (0.49–0.55)	0.50 ± 0.14 (0.45–0.55)	0.55 ± 0.12 (0.51–0.59)	—	—	0.53 ± 0.12 (0.47–0.58)	0.50 ± 0.15 (0.42–0.57)	0.56 ± 0.14 (0.50–0.62)	—	—	—	—
TV A, m/s	0.33 ± 0.11 (0.29–0.37)	0.44 ± 0.12 (0.40–0.49)	0.31 ± 0.07 (0.29–0.34)	**0.020**	>0.05	0.35 ± 0.16 (0.28–0.42)	0.48 ± 0.13 (0.41–0.54)	0.34 ± 0.15 (0.27–0.42)	**0.038**	>0.05	—	—
TV E/A	1.72 ± 0.45 (1.54–1.89)	1.15 ± 0.24 (1.06–1.24)	1.80 ± 0.38 (1.66–1.94)	**<0.001**	>0.05	1.62 ± 0.45 (1.43–1.82)	1.08 ± 0.35 (0.91–1.26)	1.79 ± 0.66 (1.47–2.11)	**<0.001**	>0.05	—	—
TV DT, ms	200 ± 62 (176–224)	166 ± 49 (147–185)	217 ± 68 (191–243)	**0.025**	>0.05	202 ± 31 (188–216)	163 ± 43 (138–187)	197 ± 32 (183–211)	**0.010**	>0.05	—	—

SD, standard deviation; CI, confidence interval; MV e’, average peak early diastolic tissue velocity of mitral valve; MV A, peak atrial flow velocity of mitral valve; MV DT, deceleration time of peak early velocity of the mitral valve; MV E, peak early flow velocity of mitral valve; TV A, peak atrial flow velocity of tricuspid valve; TV DT, deceleration time of peak early velocity of the tricuspid valve; TV E, peak early flow velocity of tricuspid valve. Statistically significant values are marked with bold.

### 3.3 Biochemical analysis

**FIGURE 3:**
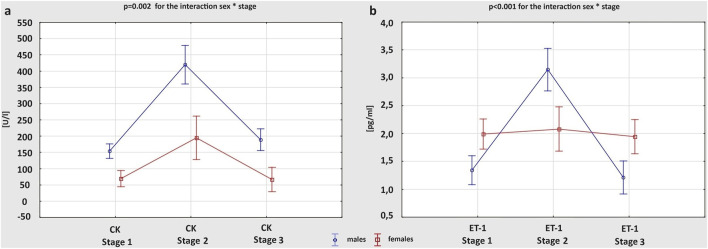
Changes in the concentration of cardiac biomarkers: creatine kinase (CK) **(A)** and endothelin-1 (ET-1) **(B)**, between three study stages in male and female amateur marathon runners.

Table 4 summarizes changes of cardiac biomarkers’ concentrations in male and female athletes, depending on the stage of the study. There were no significant differences between Stage 1 and Stage 3 in any of the obtained measurements. After the marathon run, we observed a significant increase in the level of all cardiac biochemical parameters in both males and females, except for ET-1 and neopterin, which increased post-race only in males. Both at Stage 1 and 2 the mean concentration of CK was higher in men than in women, and the exercise-induced increase in CK level was greater in males; p = 0.002 for the interaction sex * stage (Figure 3A). The concentrations of hs-TnI, H-FABP and BNP did not differ significantly between sexes at any stage of the study. However, more female amateur athletes than men met the cut-off value of hs-TnI for myocardial ischemia (>0.0342 ng/mL) at Stage 2 (70.4% vs 42.8%; p = 0.031). After the competition, female runners had remarkably higher Gal-3 levels than males, while IL-6 levels were greater in women both at rest and after the race. Although, the concentration of ET-1 at baseline was higher in females, at Stage 2 it was greater in males. Consequently, the post-race increment of ET-1 level was more pronounced in men than in women; p < 0.001 for the interaction sex * stage (Figure 3B). The concentration of neopterin was greater in males at all stages of the study.

**TABLE 4:** Concentrations of cardiac biomarkers in male and female amateur athletes.

Parameter	Male (N = 34)					Female (N = 27)					Males vs. Females	
	Stage 1	Stage 2	Stage 3	S1 vs. S2 p-value	S1 vs. S3 p-value	Stage 1	Stage 2	Stage 3	S1 vs. S2 p-value	S1 vs. S3 p-value	Stage 1	Stage 2
	Mean ± SD (95% CI)					Mean ± SD (95% CI)					p-value	
CK, U/L	150 ± 76 (122–178)	417 ± 166 (357–477)	182 ± 111 (141–223)	**<0.001**	>0.05	80 ± 40 (64–96)	211 ± 138 (156–267)	70 ± 36 (55–86)	**<0.001**	>0.05	**<0.001**	**<0.001**
hs-cTnI, ng/mL	0.01 ± 0.01 (0.00–0.01)	0.06 ± 0.09 (0.03–0.10)	0.00	**0.001**	>0.05	0.02 ± 0.04 (0.00–0.03)	0.12 ± 0.17 (0.05–0.18)	0.01 ± 0.01 (0.00–0.02)	**0.003**	>0.05	—	—
H-FABP, ng/mL	2.07 ± 1.05 (1.41–2.74)	13.42 ± 9.64 (9.83–17.01)	1.38 ± 0.67 (1.13–1.64)	**<0.001**	>0.05	1.66 ± 0.90 (1.30–2.01)	13.30 ± 7.66 (10.28–16.33)	1.47 ± 0.67 (1.18–1.75)	**<0.001**	>0.05	—	—
BNP, pg/mL	77 ± 45 (57–97)	154 ± 166 (84–224)	94 ± 47 (76–113)	**0.032**	>0.05	86 ± 55 (40–133)	218 ± 141 (140–296)	90 ± 87 (10–170)	**0.034**	>0.05	—	—
Gal-3, ng/mL	8.65 ± 2.97 (7.54–9.76)	10.80 ± 2.18 (9.99–11.61)	8.82 ± 1.65 (8.21–9.44)	**0.002**	>0.05	8.13 ± 3.62 (6.70–9.56)	13.43 ± 4.23 (11.75–15.10)	7.42 ± 2.45 (6.39–8.46)	**<0.001**	>0.05	—	**0.004**
ET-1, pg/mL	1.29 ± 0.33 (1.16–1.41)	3.19 ± 0.90 (2.84–3.53)	1.19 ± 0.26 (1.10–1.30)	**<0.001**	>0.05	1.95 ± 0.81 (1.63–2.27)	2.06 ± 0.90 (1.69–2.42)	1.87 ± 1.07 (1.42–2.32)	—	—	**<0.001**	**<0.001**
IL-6, pg/mL	1.01 ± 0.76 (0.50–1.52)	33.20 ± 13.37 (28.13–38.28)	1.52 ± 1.90 (0.26–2.79)	**<0.001**	>0.05	4.05 ± 5.04 (1.82–6.28)	50.30 ± 27.22 (39.53–61.06)	6.11 ± 1.07 (2.81–9.41)	**<0.001**	>0.05	**0.047**	**0.004**
Neopterin, nmol/L	8.36 ± 2.68 (7.36–9.36)	9.92 ± 2.14 (9.10–10.75)	7.84 ± 1.94 (7.10–8.57)	**0.003**	>0.05	5.08 ± 2.67 (4.01–6.16)	4.84 ± 2.69 (3.75–5.93)	4.88 ± 2.95 (3.57–6.19)	—	—	**<0.001**	**<0.001**

SD, standard deviation; CI, confidence interval; BNP, B-type natriuretic peptide; CK, creatine kinase; ET-1, endothelin-1; Gal-3, galectin-3; H-FABP, heart-type fatty acid binding protein; hs-cTnI, high sensitivity cardiac troponin I; IL-6, interleukin-6. Statistically significant values are marked with bold.

**FIGURE 4:**
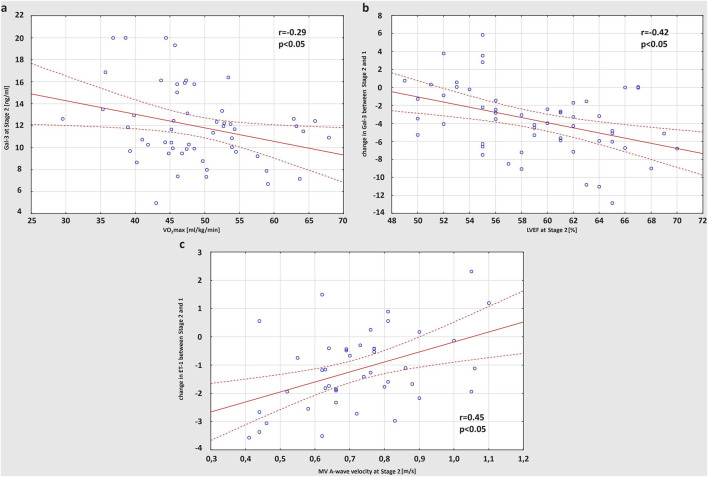
Correlation between VO_2_max (maximal oxygen uptake) and concentration of Gal-3 (galectin-3) obtained at Stage 2 **(A)**, post-run LVEF (left ventricular ejection fraction) and the increase in Gal-3 concentration after the race **(B)**, A-wave velocity of MV (mitral valve) obtained at Stage 2 and the post-marathon increase in ET-1 (endothelin-1) level **(C)**.

The correlations between basic characteristics and training habits of the study participants have been previously presented (Lasocka et al., 2021). Both distance and hours of training per week were positively related to the age of amateur athletes (r = 0.35 and r = 0.47, respectively), while the average marathon race time achieved on the finish line was negatively correlated to VO_2_max (r = 0.52) (Lasocka et al., 2021). Based on the outcomes from the current research, less trained runners with lower VO_2_max values showed higher levels of Gal-3 at Stage 2 (r = −0.29) (Figure 4A). Including echocardiographic measures in the analysis, there was a positive correlation between RVEF and LVEF values after the marathon (r = 0.39). Moreover, runners with a greater drop in LVEF at Stage 2 had larger rise in Gal-3 concentrations after the race (r = −0.42) (Figure 4B). Also, a strong positive correlation was found between A-wave velocity of MV obtained post-marathon and exercise-induced increase in ET-1 level (r = 0.45) (Figure 4C).

Table 5 presents the observed correlations between the studied biomarkers obtained after the marathon run. We found a strong positive correlation between the concentrations of ET-1 and CK (r = 0.41), as well as ET-1 and neopterin (r = 0.54). Neopterin was also positively related to CK (r = 0.48). The amateur athletes with higher post-race hs-cTnI levels presented with elevated concentrations of both Gal-3 and H-FABP at Stage 2 (r = 0.37 and r = 0.32, respectively). Finally, exercise-induced H-FABP levels correlated positively with the CK concentrations (r = 0.49). No statistically significant and strong correlations were observed between the other biomarkers collected at the marathon finish line.

**TABLE 5:** Correlations between the studied biomarkers at stage 2 in the whole study group.

	CK	Hs-cTnI	H-FABP	BNP	Gal-3	ET-1	IL-6
Neopterin	**0.48** ^a^	−0.17	0.13	−0.35	−0.24	**0.54** ^b^	−0.18
IL-6	−0.08	−0.04	0.22	0.05	0.18	−0.14	
ET-1	**0.41** ^c^	−0.04	0.15	0.07	−0.24		
Gal-3	−0.00	**0.37** ^d^	0.24	−0.07			
BNP	−0.00	0.03	−0.05				
H-FABP	**0.49** ^e^	**0.32** ^f^					
hs-cTnI	0.06						

^a^ p < 0.001.

^b^ p < 0.001.

^c^ p = 0.002.

^d^ p = 0.005.

^e^ p < 0.001.

^f^ p = 0.014.

BNP, B-type natriuretic peptide; CK, creatine kinase; ET-1, endothelin-1; Gal-3, galectin-3; H-FABP, heart-type fatty acid binding protein; hs-cTnI, high sensitivity cardiac troponin I; IL-6, interleukin-6. Statistically significant values are marked with bold.

## 4 Discussion

The current study provides a comprehensive assessment of morphological, functional and biochemical changes in amateur athletes’ heart, induced by acute bout of endurance exercise. To the best of our knowledge this is the first research that compares exercise-induced cardiac remodelling between male and female amateur marathon runners, with the use of modern echocardiographic techniques and novel cardiac biomarkers. We proved that completing a marathon resulted in transient increase in RV dimensions and concomitant reduction in LV volumes, with statistically significant functional alterations. Biochemical analysis revealed also acute elevation of the studied parameters that normalized within the detraining period. Moreover, less trained amateur athletes showed greater reaction to the same bout of exercise than those with higher VO_2_max values, in the form of greater post-run increase in biomarkers’ concentrations. Finally, significant correlations were found between echocardiographic functional measures and level of cardiac biomarkers in response to endurance training.

Although cardiovascular system has been extensively evaluated with the use of two-dimensional (2D) echocardiography also among amateur athletes, the 3D method has gained in importance over the past few decades. This advanced technique improves the diagnostic accuracy of cardiac ultrasound, allowing similar quantification of biventricular morphology and function to cardiac magnetic resonance but with the advantage of being more reproducible, economical and applicable to a larger population (De Castro et al., 2006). There are several reports based on 3D echocardiographic assessment of cardiac adaptation to training (Caselli et al., 2011; D'Andrea et al., 2003). However, these studies determine sex-specific differences between athletes only under resting conditions, without estimation of the acute changes, as presented in our research.

We noticed larger indexed LV volumes in females compared to males at baseline and after the run. As RV size did not differ significantly between sexes at any stage of the study, RVEDV/LVEDV ratio was higher in men. These outcomes correspond with previous report by D’Ascenzi et al. (2020) on 720 Olympic athletes, in which women had larger ventricular dimensions indexed to BSA and relatively higher LV/RV ratios than men. It means that female sex might predispose to greater LV plasticity and, consequently, greater capacity for cavity enlargement. Additionally, we found that resting parameters of biventricular systolic function were greater in female amateur runners than males, regardless of the study stage. Fábián et al. (2022), who analysed 422 elite althletes of both sexes, confirmed this observation and revealed mild but statistically significant reduction in LVEF and RVEF, accompanied by a decrease in global strain rates in males compared to females.

Analysing the acute changes, after the marathon we observed a transient increase in RV volumes with concomitant decrease in LV cavity. The observation was compatible in male and female group. Moreover, the RV contractility decreased post-race in both study groups, while LV systolic function remained unchanged. In response to ultra-endurance training, Dávila-Román et al. (1997) also described marked RV dilatation and reduction in function, without any signs of LV damage. Considering this ventricular interdependence, La Gerche et al. (2011) reported a relatively greater increase in pulmonary pressure during endurance exercise compared to systemic blood pressure and, consequently, greater involvement of RV than LV. Indeed, it is likely the acute rise in RV afterload that induce secondary changes in ventricular morphology and function (Douglas et al., 1990a). Along with RV remodelling, both septum and LV apex might be incidentally affected giving the impression of LV dysfunction. Indeed, we found a positive correlation between post-race decrease in RVEF and LVEF values. There are several other reports suggesting LV functional augmentation following prolonged training (Douglas et al., 1990b; Vanoverschelde et al., 1985). However, it is thought to be highly dependent on the exercise load and environmental conditions (Douglas et al., 1987). Analysing sex-specific discrepancies, McGavock et al. (2003) confirmed the exercise-induced reduction in LV size with preserved systolic function in the population of female triathletes. As presented in our research, men are though more prone to develop a transient decrease in LV contractility, which might be explained by dominant sympathetic regulation both at rest and during exercise in male athletes compared to parasympathetic predominance in females (Scott et al., 1985). Consequently, the increased sympathetic response might result in a greater beta-receptor responsiveness reduction and a greater decrease in LV function (Davis et al., 2000).

Apart from systolic function assessment, the evaluation of ventricular filling and myocardial relaxation should not be omitted during routine echocardiographic examination in athletes. Early impairment of diastolic function indicates certain pathologic conditions that may mimic the morphologic characteristics of the athlete’s heart. Our investigation revealed no abnormalities among the indexes of biventricular diastolic pattern, however, endorsed presence of significant sex-specific differences. Both at rest and after the run female amateur athletes had higher E− and A-waves’ velocities of MV. Although e’ values did not differ remarkably between sexes, E/e’ ratios were increased in women. Caselli et al. (2015) confirmed these observations in the large cohort of male and female Olympic athletes, indicating greater filling pressures among women. These seem to be dictated by alterations in LV geometry that are considered more pronounced in men (Wooten et al., 2021). On the contrary, the RV diastolic measures did not differ remarkably between sexes at any stage of the study, what corresponded with previous research by Zaidi et al. (2013), who assessed RV adaptation to training depending on ethnicity and sex.

Completing a marathon resulted in the impairment of diastolic function in males and females, manifested by E-wave velocities reduction, A-wave velocities increase and, consequently, significantly decreased E/A ratios. Several other investigations on amateur marathon runners of both sexes have reported such changes in LV filling, even with preserved systolic function (Lucía et al., 1999; George et al., 2004). In our study the transient decrease in E/A ratio could not be explained by raised HR after the run due to lack of correlation between these parameters. Exercise-induced LV overload in the form of increased concentrations of wall stress markers could have contributed to the observed diastolic dysfunction. Indeed, we noticed a strong positive correlation between A-wave velocity of MV at Stage 2 and post-marathon increase in ET-1 level, which expression was proved to augment in response to myocardial stretching during diastole (Loennechen et al., 2001). Moreover, we observed a statistically significant post-race reduction in e’ values, without alterations in E/e’ ratios, in male and female groups. However, female athletes experienced more pronounced tendency for the growth of E/e’ ratio; p = 0.040 for the interaction sex * stage. George et al. (2005) achieved similar outcomes in his study on London Marathon participants of both sexes, proving that the exercise-induced depression in diastolic function is likely due to altered relaxation of the left ventricle. While Knebel et al. (2014) analysed the influence of marathon on pre- and postmenopausal female runners and reported a significant post-race increase of E/e’ ratio regardless of the serum hormone pattern, but greater decrease of E/A ratio in the postmenopausal group reflecting the protective role of female steroid hormones.

Biochemical analysis of the study participants revealed corresponding changes in the level of cardiac biomarkers. When focusing on myocardial necrosis enzymes, we noticed a remarkable increase in the CK and hs-TnI levels following the marathon run, both in male and female amateur athletes. However, the post-race elevation in CK was greater in males than in females, and men had higher mean concentrations of CK than women at each stage of the study. Indeed, there are many reports supporting the short-term increase in cardiac injury markers, also in the heterogenous group of non-elite athletes (Jassal et al., 2009; Kratz et al., 2002). Jassal et al. (2009) examined 121 amateur individuals of both sexes completing either half-marathon or full marathon, and demonstrated elevated values of CK and hs-TnI after the run that positively correlated with the increasing training time. As levels of the presented enzymes are thought to normalize within detraining period, this transient biomarker release should be treated rather as physiological response than real myocardial injury. Previous studies on healthy marathon runners and cyclists with positive values of cardiac necrosis enzymes after endurance exercise excluded myocardial damage or dysfunction with the use of modern imaging technics (Siegel et al., 2001; Scharhag et al., 2006).

Comparing males to females, according to Shumate et al. (1979) women exhibit significantly lower serum CK levels than men, and the sex discrepancy is even larger after exercise, what corresponds with our outcomes. It might be explained by sex-specific variations in muscle fiber recruitment or in muscle mass, along with protective effect of estrogen on exercise-induced muscle injury in females (Rogers et al., 1985). Although similar sex differences were reported for hsTnI (Kong et al., 2017; Legaz-Arrese et al., 2017), we found no alterations in either resting or post-race concentrations of hsTnI between men and women. In addition, more female athletes met cut-off criteria for myocardial ischemia at Stage 2 than males. This may be partially explained by an adaptive process occurring in the heart due to greater exercise load in the form of training time per week and, consequently, greater LV remodelling detected in female group. Several researchers confirmed the positive correlation between training level and hsTnI release (Legaz-Arrese et al., 2015; Saravia et al., 2010), however, conflicting data exist in the literature and future work should evaluate the possible hypotheses.

Another early marker of myocardial damage is H-FABP. Although rarely applied in the screening of athletes, H-FABP next to hs-TnI and CK possesses high diagnostic sensitivity for acute myocardial infarction (Chen et al., 2004). In the previous studies on marathon runners and cyclists, acute hemodynamic changes associated with a prolonged training resulted in transient increase in H-FABP level (Scherr et al., 2011; Williams et al., 2011; Żebrowska et al., 2019). Our research revealed similar outcomes in the group of amateur athletes, without statistically significant differences between sexes. In addition, we found positive correlations between post-race H-FABP concentration and exercise-induced activity of both hsTnI and CK. According to Żebrowska et al. (2010), due to different release times and lower times of reaching maximal concentrations from other markers of myocardial injury, H-FABP may be more effective in detecting post-exercise cardiac damage. However, early release of H-FABP in response to training might be explained by its lower molecular mass and higher membrane permeability than CK or hs-TnI, and thus should be considered rather physiological.

Moreover, endurance exercise, such as marathon running, promotes transient myocardial wall stress responsible for increased secretion of BNP by cardiomyocytes. As a counter-regulatory hormone, BNP induces natriuresis, diuresis and vasodilation, reducing cardiac pressure and volume overload connected with the prolonged training (Scharhag et al., 2008). Several reports on healthy athletes of both sexes demonstrated post-exercise BNP elevation (Frassl et al., 2008; Siegel et al., 2001; Ohba et al., 2001). We also noticed a statistically significant increase in the BNP concentration after the marathon, in both male and female amateur runners. Although the BNP level did not differ remarkably between sexes at any stage of the study, women are prone to develop higher values of the biomarker due to positive effect of estrogen on the synthesis of cardiac natriuretic peptides (Clerico et al., 2019).

Gal-3 represents a group of markers of cardiac remodelling and fibrosis released in response to myocardial stretch and inflammatory processes by activated macrophages. Increased Gal-3 levels were proved to predict outcome in heart failure patients and all-cause mortality in the general population (de Boer et al., 2012; Hrynchyshyn et al., 2013). Besides, our study revealed that exercise-induced myocardial stretch and inflammation results in transient elevation in Gal-3 concentration in male and female amateur athletes. This observation corresponds with few other reports on endurance runners (Hättasch et al., 2014; Le Goff et al., 2020). However, the extent of increase was greater in female counterparts, which could be at least partially explained by larger training dose a week in women. Post-race Gal-3 level showed a negative correlation with the training experience (VO_2_max in CPET) of the study participants, meaning that those who were less fit had greater response to the same bout of exercise. This could be an adaptive mechanism to repetitive training, possibly with positive impact on long-term outcomes. What is more, we noticed that amateur athletes with lower post-race LVEF values had greater increase in Gal-3 after the run, suggesting that effort-induced cardiac fibrosis may induce short-term LV dysfunction. Similar negative correlation was previously described by Lewicka-Potocka et al. (2022), however, it concerned relationship between biomarkers’ level and contractility of RV, which is considered more susceptible to hemodynamic changes associated with prolonged training than LV. Finally, increased Gal-3 concentrations on the finish line were positively related to the post-run hs-TnI elevation. Le Goff et al. (2019) explained that ischemia might induce inflammatory response and, consequently, macrophage infiltration responsible for development of cardiac fibrosis.

Furthermore, we observed a post-race increase in ET-1 levels of amateur athletes, more pronounced in male than female marathon runners. ET-1 is not only a potent vasoconstrictor released by vascular endothelial cells, but also has positive inotropic and chronotropic effect on myocardium, inducing cardiac hypertrophy (Shubeita et al., 1990). As mechanical stretching of cardiomyocytes is associated with an increase in ET-1 expression (Yamazaki et al., 1996), it might be hypothesised that exercise-induced hemodynamic overload influences ET-1 production. Indeed, in the study on rat models, Maeda et al. (1998) reported that prolonged exercise caused elevation in the circulating ET-1 concentration. Conflicting data exist on sex-specific ET-1 levels. According to Polderman et al. (1993) men have higher ET-1 concentrations than women thanks to protective effect of female steroid hormones. However, other study on healthy human volunteers of both sexes suggested that estrogen is responsible for ET-1 plasma elevation and, consequently, revealed greater ET-1 levels in women (Evans et al., 1996), which corresponded with our observations at baseline. While after the marathon run, we found significantly higher ET-1 concentrations in male athletes compared to females. This could be explained by greater sympathetic nervous system drive during exertion in men than in women, which function is thought to be regulated by endothelin signalling (Lehmann et al., 2014). Nevertheless, the differences between sexes remain unexplored due to lack of research on athletes, and serve as a foundation for future investigation.

Prolonged exercise induces oxidative stress due to an increased need for endogenous fuel mobilization. That upregulates inflammatory response and stimulates cytokines’ production (Nash et al., 2023). IL-6 belongs to the group of proinflammatory cytokines, responsible for fatty acid oxidation and lipolysis of fat stores under conditions of increased demand for metabolic substrate availability. Our research showed that marathon run promotes IL-6 elevation in both male and female amateur athletes, which is consistent with previous findings of Scherr et al. (2011)) and Pinho et al. (2010). Although it has been reported that women are less susceptible to oxidative stress and, consequently, have lower concentrations of oxidative stress markers due to their higher activity of antioxidant estrogen (Fisher-Wellman and Bloomer, 2009), we noticed higher IL-6 levels in the female group regardless of the study stage. The uncertain hormonal profile and greater training load among women could justify the observed difference in inflammatory response between sexes.

Finally, neopterin is a clinical marker of cellular immune activation, released by human monocyte-derived macrophages and dendritic cells upon stimulation by gamma-interferon during various infectious processes (Pingle et al., 2008). Similarly to the reaction of proinflammatory cytokines, neopterin’s level was found to increase following endurance training (Schobersberger et al., 2000; Moser et al., 2008). We also noticed elevated concentration of neopterin after marathon run in male amateur athletes, without statistically significant exercise-induced changes in the female group. What is more, women had lower values of the biomarker than men, both at rest and post-race. This might be explained by larger autonomic response to acute stress and, consequently, greater stress-induced increase in the production of the inflammatory agents in males than females (Klein and Flanagan, 2016). In addition, post-run neopterin level positively correlated with training-related concentrations of CK and ET-1, meaning that immune activation following endurance exercise corresponds with myocardial damage and sympathetic stimulation (Sprenger et al., 1992).

Several limitations of the study should be noticed. Firstly, the study was carried out in a relatively small sample and involved only white participants living in the Pomeranian Voivodeship, Poland, limiting its statistical power and applicability. Secondly, lack of a control group of non-amateur marathon runners matched by sex, age, height, and weight precludes confident correlation of gender differences with endurance exercise. In addition, male and female amateur athletes were not matched by the training status, which could have at least partially influenced the obtained results. What is more, female participants might have been divided into pre- and postmenopausal groups, as hormonal status is a significant predictor of cardiac response to training. Finally, the Stage 2 of the study was conducted directly after endurance exercise, which cannot fully extrapolate to the acute exertion phase.

## 5 Conclusion

To the best of our knowledge this is the first study that assess echocardiographic changes and biochemical results in the group of amateur athletes, focusing on sex-specific differences. Completing a marathon affected mainly RV in the form of chamber dilatation and decrease in function, with less prominent remodelling on the left side of the heart. The echocardiographic alternations corresponded with the elevation of biomarkers of cardiac injury and overload. As the observed changes were transient and returned to baseline values within 2 weeks after the run, they should be related rather with physiological myocardial response to acute bout of endurance exercise than actual myocardial damage. However, it can-not be omitted that the population of endurance athletes, especially amateurs, is heterogenous in terms of lifestyle or cardiovascular risk factors, and the same training load may differently affect two unrelated sportsmen.

Our research also confirmed specific sex-related direction of cardiac adaptation to exercise. Females had larger LV volumes both at rest and after the run, proving predominant eccentric remodelling in this sex group. On the contrary, men presented with greater tendency for LV contractility reduction following training, which positively correlated with the post-race Gal-3 level, suggesting possible link between cardiac muscle alteration and transient LV dysfunction. What is more, concentrations of the majority of the studied biomarkers, including parameters of myocardial damage and the proinflammatory agents, were significantly higher in male than female athletes. Considering these structural, functional and biochemical differences, men appear to develop more pronounced cardiac remodelling in response to endurance training than women. As the observed myocardial changes potentially constitute arrhythmia triggers, it is not surprising that male athletes have higher risk of malignant heart rhythm disturbances along with greater incidence of SCD.

To conclude, both echocardiographic examination and biochemical blood tests next to ECG constitute non-invasive techniques that enable comprehensive assessment of the athlete’s heart. Recognising physiological cardiac adaptation to training in males and females is of clinical importance, as certain phenotypes may mimic underlying structural heart diseases. We would recommend cardiovascular screening not only in elite but also in amateur athletes in daily practice so as to identify subjects requiring further evaluation.

## Data Availability

The raw data supporting the conclusions of this article will be made available by the authors, without undue reservation.
